# Protective Effects of Costunolide against Hydrogen Peroxide-Induced Injury in PC12 Cells

**DOI:** 10.3390/molecules21070898

**Published:** 2016-07-09

**Authors:** Chong-Un Cheong, Ching-Sheng Yeh, Yi-Wen Hsieh, Ying-Ray Lee, Mei-Ying Lin, Chung-Yi Chen, Chien-Hsing Lee

**Affiliations:** 1 Department of Intensive Care Medicine, Chi Mei Medical Center, Liouying, Tainan City 73657, Taiwan; jhnchong@me.com; 2 Department of Nutrition and Health Science, School of Medical and Health Sciences, Fooyin University, Kaohsiung City 83102, Taiwan; janson.yeh@msa.hinet.net; 3 Bio-Medical Technology Developmental Center, Fooyin University, Kaohsiung City 83102, Taiwan; 4 Department of Medical Technology, School of Medical and Health Sciences, Fooyin University, Kaohsiung City 83102, Taiwan; 5 Department of Medical Research, Fooyin University Hospital, Ping-Tung County 92847, Taiwan; 6 Department of Mackay Memorial Hospital Taitung Branch, Taitung County 95054, Taiwan; yisnolly@gmail.com; 7 Department of Medical Research, Chiayi Christian Hospital, Chiayi City 60002, Taiwan; yingray.lee@gmail.com; 8 Cancer Center, Kaohsiung Medical University Hospital, Kaohsiung City 80708, Taiwan; eileen26854@yahoo.com.tw; 9 Department of Nursing, Min-Hwei Junior College of Health Care Management, Tainan City 73658, Taiwan

**Keywords:** reactive oxygen species, costunolide, PC12 cells, mitochondria membrane potential

## Abstract

Oxidative stress-mediated cellular injury has been considered as a major cause of neurodegenerative diseases including Alzheimer’s and Parkinson’s diseases. The scavenging of reactive oxygen species (ROS) mediated by antioxidants may be a potential strategy for retarding the diseases’ progression. Costunolide (CS) is a well-known sesquiterpene lactone, used as a popular herbal remedy, which possesses anti-inflammatory and antioxidant activity. This study aimed to investigate the protective role of CS against the cytotoxicity induced by hydrogen peroxide (H_2_O_2_) and to elucidate potential protective mechanisms in PC12 cells. The results showed that the treatment of PC12 cells with CS prior to H_2_O_2_ exposure effectively increased the cell viability. Furthermore, it decreased the intracellular ROS, stabilized the mitochondria membrane potential (MMP), and reduced apoptosis-related protein such as caspase 3. In addition, CS treatment attenuated the cell injury by H_2_O_2_ through the inhibition of phosphorylation of p38 and the extracellular signal-regulated kinase (ERK). These results demonstrated that CS is promising as a potential therapeutic candidate for neurodegenerative diseases resulting from oxidative damage and further research on this topic should be encouraged.

## 1. Introduction

Reactive oxygen species (ROS), a substance resulting from neuronal injury during oxidative stress, regulates many cellular activities under physiological conditions [1]. Oxidative stress- mediated cellular injury has long been associated with a variety of neurodegenerative diseases such as Alzheimer’s disease, Parkinson’s disease, stroke, and amyotrophic lateral sclerosis [2,3,4,5]. Furthermore, H_2_O_2_ is thought to be the major precursor of ROS and has been utilized extensively as an inducer of oxidative damage to interpret mechanisms and the neuroprotective potential of therapeutics [6].

Several evidences have showed that H_2_O_2_ induces cytotoxicity in rat pheochromocytoma (PC12), which has neuron-like characteristics and provides a useful model system in analyzing the neurological apoptosis and the prevention mechanisms of antioxidants [7,8,9]. Moreover, H_2_O_2_-induced apoptosis has been linked to various key alterations including in anti-apoptosis proteins, pro-apoptosis proteins and caspases. Therapeutic strategies focusing on prevention of the ROS mediated by antioxidants seem to have potential for delaying the diseases’ progression [10]. Many synthetic antioxidants have been demonstrated to be strong radical scavengers, but they are also carcinogenic and cause liver damage [11]. Therefore, much attention has recently been focused on the isolation and identification of antioxidants from natural sources with neuroprotective potential [12,13]. 

**Figure 1:**
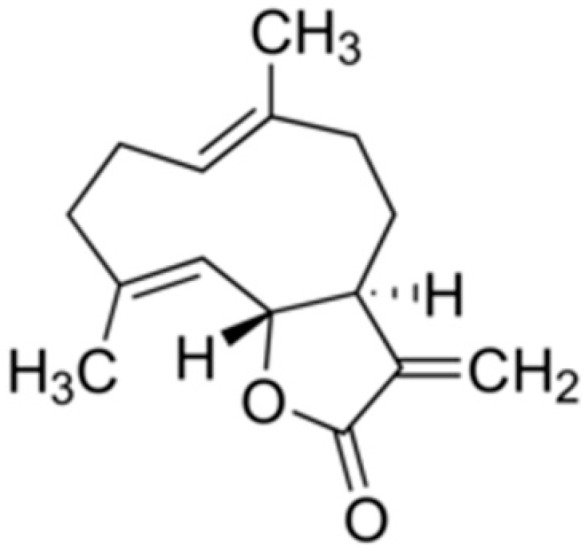
Chemical structure of costunolide (CS).

Costunolide (CS) is a sesquiterpene lactone found in the leaves of *Laurus nobilis* (Lauraceae), which has been reported to have anti-inflammatory [14], neuronal dopaminergic cells protection [15], anti-viral and anti-fungal properties [16,17], as well as cytotoxic effects on various human cancer cells [18] and antioxidant activity [19]. The chemical structure of CS is shown in Figure 1. However, its neuroprotective activity has yet to be explored. In this study, we used H_2_O_2_-induced oxidative damage in PC12 cells as an in vitro model to determine the neuroprotective activity of CS and to further investigate the mechanism.

## 2. Results and Discussion

### 2.1. Effect of Costunolide on Viability of H_2_O_2_-Induced PC12 Cells

Overproduction of ROS causes damages to the cellular structures of neurons including lipids and membranes, proteins, and DNA [20]. The oxidative stress-induced ROS is involved in the pathophysiology of major neurodegenerative diseases such as Parkinson’s and Alzheimer’s diseases [20,21,22]. Several reports suggest therapeutic strategies focused on searching for the potential targets involved in the neuroprotection of natural compounds that can scavenge free radicals and protect cells from oxidative damage [12,13]. Previous studies have revealed that CS possesses antioxidant activities [19]. However, whether CS can exert protective effects against oxidative cytotoxicity in neuronal models as a result of its antioxidant properties has not been established in the literature.

**Figure 2:**
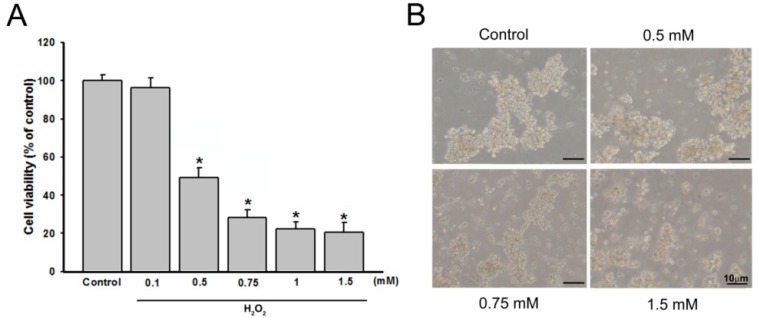
Effects of H_2_O_2_ on PC12 cell viability and cell morphology. (**A**) Effect of H_2_O_2_ on viability of PC12 cells (exposure to 4 h). A MTT assay showed that H_2_O_2_ decreased cell viability in a concentration-dependent manner; (**B**) treatments with different concentrations induced cell morphological alterations. Data were summarized from three independent experiments. * *p* < 0.05 vs*.* control group.

A pilot study revealed that H_2_O_2_ ranging from 0.1 to 1.5 mM leads to cell death in a dose dependent manner and 0.75 mM H_2_O_2_ induced cell injury in a moderate manner (Figure 2A). These morphological alterations are reported illustrated in Figure 2B. The aim of the study was to investigate the effects of antioxidants over a short time frame (0–6 h). Therefore this concentration (0.75 mM H_2_O_2_) was used for all further experiments. The high concentration of H_2_O_2_ exposure of PC12 cells is consistent with investigations of the neuroprotective effects of macranthoin G [9] and the flavonoid extracts [23].

**Figure 3:**
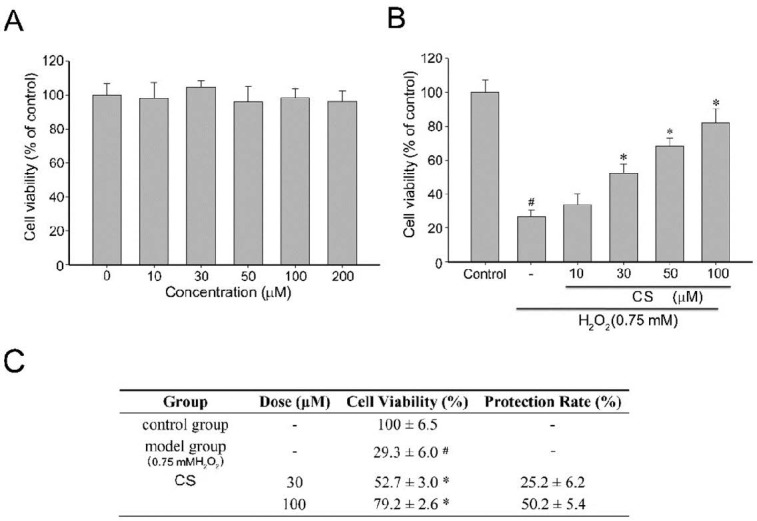
Cytotoxicity and cytoprotective activity of costunolide (CS). (**A**) PC12 cells were pretreated with various concentrations of CS for 4 h; (**B**) Cell viability of PC12 cells pretreated with CS (10, 30, 50 and 100 µM) 1 h before exposure to H_2_O_2_ (0.75 mM) 4 h was measured by the MTT assay. Data are presented as mean ± SD (*n* = 3) and (**C**) The protection rates of CS are shown. Values with the same superscript letters are not significantly different from each other. # *p* < 0.01 compared with the control group; * *p* < 0.05, compared with the model group.

To characterize the effects of CS on cell viability in the H_2_O_2_-stressed cultured PC12 cells, the cells were incubated with CS and 0.75 mM H_2_O_2_. The H_2_O_2_-induced cell death of cells was determined by MTT assays. As shown in Figure 3A, PC12 cells exposed to CS (0–200 µM) for 4 h did not exhibit any significant viability or proliferation alterations. However, incubation with 0.75 mM H_2_O_2_ for 4 h resulted in a cell viability rate of 26.9% compared to the control (Figure 3B). In contrast, pretreatment of the cells with CS (10, 30, 50, or 100 µM) for 1 h could remarkably restore cell survival to 34.0%, 55.33%, 90.8%, and 95.87%, respectively. The potency of 100 µM vitamin E was similar to that of 50 µM CS (data not shown). Moreover, the H_2_O_2_-induced neuronal injury was accompanied by changes in cell morphology as observed in the loss of the characteristic round form and grouping shaped in PC12 cells. According to the respective calculations, it was shown that the protection rates of CS were reported in Figure 3C. Results suggested that CS could be considered as a neuroprotective agent against H_2_O_2_-induced oxidative stress.

### 2.2. Effect of CS on H_2_O_2_-Induced ROS Production and Mitochondria Membrane Potential (MMP) in PC12 Cells

**Figure 4:**
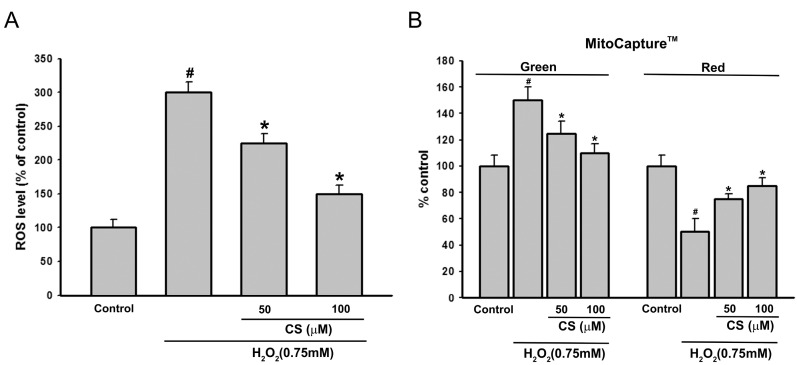
Effect of costunolide (CS) on H_2_O_2_-induced intracellular accumulation of ROS and mitochondria membrane potential (MMP). Intracellular ROS levels and MMP were measured using the MitoCapture™ Kit. PC12 cells were pretreated with various concentrations of CS for 30 min before exposure to 0.75 mM H_2_O_2_ for 6 h. (**A**) Histogram showing the ROS level in PC12 cells after treatment with H_2_O_2_ in presence or absence of CS compared to untreated groups; (**B**) Histogram showing the number of cells with a low potential in PC12 cells after treatment with H_2_O_2_ in presence or absence of CS compared to untreated groups. Cells were stained with MitoCapture™ solution, demonstrating both a reduced number of healthy cells (red signal) and increased number of cells with disrupted mitochondrial potential (green signal) in the presence of CS. Data are presented as mean ± SD (*n* = 3). Values with the same superscript letters are not significantly different from each other at # *p* < 0.01 compared with the control group; * *p* < 0.05, compared with the model group.

Oxidative stress-induced ROS production contributes to cell death by oxidation of many important proteins, leading to mitochondrial dysfunction and cell death [24,25]. To provide further evidence that CS could prevent H_2_O_2_-induced ROS generation and oxidative stress, levels of ROS production in the cells were determined using the fluorescence probe DCFH-DA for measuring the fluorescent compound dichlorofluorescein (DCF) [26]. As shown in Figure 4A, when cells were only exposed to 0.75 mM H_2_O_2_ for 6 h, the DCF fluorescence intensity increased significantly (305.18% of control group). Pretreatment with CS suppressed the fluorescence intensity in the H_2_O_2_-induced PC12 cells, suggesting that CS exerts its antioxidant effect in the intracellular compartment. A recent study indicated that the CS possessed protective effects on ethanol-induced oxidative gastrointestinal mucosal injury through restoration of oxidative stress markers, such as superoxide dismutase (SOD) and malondialdehyde (MDA) [27]. These results confirm that CS has antioxidant activity against ROS.

Excessive ROS production would damage mitochondrial membrane integrity and affect the energy production in mitochondria, resulting in mitochondrial dysfunction [28,29]. Furthermore, mitochondrial dysfunction includes a decrease in mitochondria membrane potential (MMP), activation of caspase-3, and apoptosis [30]. Therefore, we studied the effect of CS on MMP induced by H_2_O_2_ using the MitoCapture™ Apoptosis Detection Kit. The MitoCapture™ fluorescent dye was used as a marker for apoptosis. In healthy cells, the reagent congregates in the mitochondria and is detected as a red fluorescence signal. Conversely, in apoptotic cells, the dye remains in the cell cytosol (due to the disrupted mitochondrial membrane potential) and can be monitored as a green fluorescent signal [31]. Exposure of PC12 cells to H_2_O_2_ (0.75 mM) for 6 h induced a significant loss of MMP (Figure 4B), green fluorescence was increased by 45% ± 8%, while red fluorescence was decreased by 51% ± 6% (in both cases *n* = 30 and *p* < 0.001 compared with controls). Pretreatment with 50 µM CS significantly enhanced the reduction in MMP induced by H_2_O_2_, demonstrating that CS might change the occurrence of mitochondrial dysfunction after oxidative stress.

### 2.3. Effect of CS on H_2_O_2_-Induced Apoptosis in PC12 Cells

**Figure 5:**
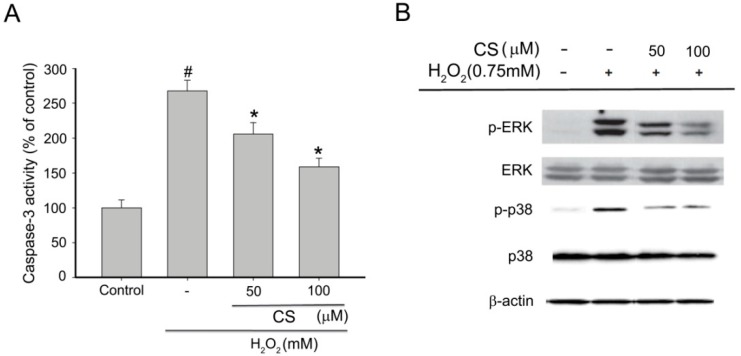
Effect of costunolide (CS) on H_2_O_2_-induced apoptosis in PC12 cells. PC12 cells were pretreated with various concentrations of MCG for 30 min before exposure to 0.75 mM H_2_O_2_ for 6 h. (**A**) The effect of CS on caspase-3 activity in H_2_O_2_-induced PC12 cells. Cells were pretreated with various concentrations of CS for 30 min before exposure to 0.75 mM H_2_O_2_ for 6 h. Caspase-3 activity was determined using a commercial kit according to the manufacturer’s instruction. Values with the same color bars with the same superscript letters are not significantly different from each other at # *p* < 0.01 compared with the control group; * *p* < 0.05, compared with the model group; (**B**) Following the same treatment, the levels of phospho- or total mitogen activated protein kinases (MAPKs) (ERK and p38) were identified by their antibodies. Results are representative of three experiments.

ROS have been demonstrated to induce damage biological molecules resulting in apoptotic or necrotic cell death [32]. Caspase-3 has been reported to be a key executioner caspase involved in neuronal apoptosis which modulates the mitochondria-dependent pathway [31]. To determine whether the cytoprotection by CS was due to the inhibition of apoptosis, the PC12 cells were treated with H_2_O_2_ and various concentrations of CS. As shown in Figure 5A, H_2_O_2_ treatment caused a remarkable increase of caspase-3 activity. However, adding 50 and 100 µM CS before H_2_O_2_ treatment decreased the caspase-3 activity to 205.68% and 158.63%, respectively. It can therefore be concluded that CS was effective in decreasing H_2_O_2_-induced apoptotic cell death. This supports the conclusion that CS inhibits H_2_O_2_-induced apoptosis through the regulation of intracellular ROS levels and mitochondria-dependent caspase-3 pathway.

### 2.4. Effect of CS on MAPK Phosphorylation in H_2_O_2_-Induced PC12 Cells

The mitogen-activated protein kinases (MAPK) signaling pathway plays an important role in cell proliferation, differentiation, and apoptosis [33,34]. They are also involved in ROS-mediated oxidative stress. ROS activates MAPKs in PC12 cells leading to apoptosis through activation of various downstream signal related events, such as MMP dissipation and activation of caspase-3 [35,36,37,38]. To further explore the effect of CS on the modulation of upstream signaling events against H_2_O_2_-stimulated oxidative stress, we examined MAPKs pathway by the immunoblot analysis. As shown in Figure 5B, pretreatment of cells with 50 and 100 µM CS significantly inhibited the H_2_O_2_-induced activation of phosphorylation of p38 MAPK and ERK. Consistent with the previous results, CS markedly inhibited LPS-induced activation of p38 MAPK and ERK [39]. A number of reports have shown that NF-κB/Rel activity is mediated by MAPKs [40]. Thus, our results provided a possible mechanism responsible for the neuroprotective effect of CS on NF-κB/Rel activity. Taken together, the inhibitory effects of CS on H_2_O_2_-induced apoptosis in PC12 cells was not only due to ROS scavenging, but also to the specific modulation of phosphorylation of p38 and ERK.

In addition, the neuroprotective effect of CS was also observed in dopamine-induced apoptosis in SH-SY5Y cells through reduction of α-synuclen [15] which increases the rate of production of ROS [41]. These results confirmed that CS is a cytoprotective agent for neurodegenerative diseases caused by ROS.

## 3. Materials and Methods

### 3.1. Chemicals and Reagents

Costunolide (CS), dimethylsulfoxide (DMSO), 1,1-diphenyl-2-picrylhydrazyl (DPPH), and 3-(4,5-dimethylthiazol-2-yl)-2,5-diphenyltetrazolium bromide (MTT) were obtained from Sigma (St. Louis, MO, USA). Dulbecco’s modified Eagle’s medium (DMEM) was purchased from Hyclone (Logan, UT, USA). Fetal bovine serum (FBS) and a penicillin/streptomycin mixture were purchased from Gibco (Grand Island, NY, USA). Vitamin E, catechin hydrate, Reactive Oxygen Species Assay Kit (S0033), cell lysis buffer for western blots and immunoprecipitation (IP) (P0013), 6× SDS-PAGE Sample Loading Buffer (P0015F) were obtained from Cell Signaling Technology (Danvers, MA, USA).

### 3.2. Cell Culture

PC12 cells were obtained from the American Type Culture Collection (ATCC), Rockville, MD, USA). PC12 cells were cultured in RPMI 1640 supplemented with 5% heat-inactivated FBS, 10% HS, 100 U/mL of penicillin, and 100 μg/mL of streptomycin. Cells were incubated at 37 °C in a humidified atmosphere of 95% air and 5% CO_2_.

### 3.3. Cell Viability Assay

Cytoprotective activity of CS on H_2_O_2_-induced cell injury was investigated by an MTT assay. The PC12 cells were seeded into 96 well plates at a density of 5 × 10^4^ cells/well for 16 h and then pretreated with vehicle alone or different concentrations of CS for 30 min before exposure to 0.75 mM H_2_O_2_ for 6 h. After removing the supernatant of each well, a total of 10 µL of MTT solution (5 mg/mL in phosphate-buffered saline (PBS)) and 90 µL of FBS-free medium were added to each well at the time of incubation for 4 h at 37 °C. The dark blue formazan crystals formed inside the intact mitochondria were solubilized with 100 μL of MTT stop solution (containing 10% sodium dodecyl sulfate (SDS) and 0.01 M hydrochloric acid). The amount of MTT formazan was determined based on the adsorption at 550 nm in a microplate reader (SpectraMax 250, Molecular Devices Inc., Sunnyvale, CA, USA). The optical density of formazan formed in control cells was taken as 100% viability. The protection rate of tested compounds was calculated using the following equation/protection rate (%) = (cell viability of drug group − cell viability of model group)/(cell viability of control group − cell viability of model group) × 100%.

### 3.4. Measurement of ROS

Generation of intracellular ROS was detected using a ROS-sensitive fluorescent probe (DCFH-DA). DCFH-DA is oxidized to highly fluorescent dichlorofluorescein (DCF) in the presence of ROS, which is readily detected by a flow cytometry [25]. A total of 1 × 10^5^ PC12 cells was plated per well in 6 well plates with 2 mL culture medium for 16 h for stabilization and exposed to CS for 30 min before exposure to 0.75 mM H_2_O_2_ for 6 h. The cells were detached by gentle pipetting and washed with PBS. Cells were treated with 20 µM DHFH-DA for 30 min in the dark at room temperature. After DCFH-DA was removed, the cells were rinsed by PBS and collected in 15 mL centrifuge tubes by gentle centrifugation. Then, the supernatants were aspirated and cell pellets re-suspended in 1 mL PBS. Cells were finally transferred to flow cytometry tubes and intracellular ROS was measured via flow cytometry (Becton-Dickinson, Franklin Lakes, NJ, USA) at an excitation wavelength of 498 nm and an emission wavelength of 522 nm. The mean fluorescent intensity (MFI) of 10,000 cells was analyzed using three replicates for each experimental condition.

### 3.5. Measurement of Mitochondrial Transmembrane Potential

Mitochondrial transmembrane potential was evaluated with a MitoCapture™ Mitochondrial Apoptosis Detection kit (MBL, Nagoya, Japan), according to the manufacturer’s instructions. CS were subjected to assays at 4 h. Images were taken under a fluorescence microscope using a bandpass filter to detect FITC and rhodamine. The cells with many aggregates giving off a bright red fluorescence represented those with a intact mitochondrial transmembrane potential and were enumerated.

### 3.6. Measurement of Caspase-3 Activity

Caspase-3 activity was measured using a commercial kit (Beyotime, Haimen, China) according to manufacturer’s instruction. Briefly, a total of 1 × 10^6^ cells was plated per well in 6 well plates with 2 mL culture medium for 16 h and exposed to CS for 30 min before exposure to 0.75 mM H_2_O_2_ for 6 h. Cells were harvested, washed twice with cold PBS and resuspended in lysis buffer on ice for 4 min. Next, cell lysates were centrifuged at 10,000× *g* at 4 °C for 10 min. Caspase-3 activity was measured by using reaction buffer and optical density was determined based on the adsorption at 405 nm using reaction buffer and optical density was determined based on the adsorption at 405 nm using an Infinite M200 Pro spectrophotometer (Tecan, Männedorf, Switzerland).

### 3.7. Data Analysis

All tests were carried out in triplicate (*n* = 3). The data are expressed as the mean ± standard derivation (SD). One-way analysis of variance (ANOVA) was used to determine the significant differences between the groups followed by a Dunnett’s t-test for multiple comparisons. A probability <0.05 was considered as significant. All analyses were performed using SPSS for Windows 7, version 19.0 (IBM Corp., New York, NY, USA).

## 4. Conclusions

The neuroprotective effect of CS against H_2_O_2_-induced apoptosis in PC12 cells was investigated. It was found that CS can decrease H_2_O_2_-induced oxidative stress in PC12 cells by decreasing the ROS level and elevating MMP as well as restoring the mitochondria-dependent caspase-3 pathway. Furthermore, CS possibly affects upstream regulatory elements, such as p38, and ERK to attenuate the oxidative stress injury induced by H_2_O_2_ in PC12 cells. CS has been proved to possess aqueous solubility, good intestinal absorption and blood-brain barrier penetration [42]. These results demonstrate the potential of CS, providing a basis for further studies on its application to combat neurologic diseases, despite the fact that CS can be quite cytotoxic against other cells so its use to combat neurological disorders would probably be quite limited.

## Abbreviations

ROS: Reactive oxygen species
CS: Costunolide
H_2_O_2_: Hydrogen peroxide
MMP: Mitochondria membrane potential
ERK: Extracellular signal-regulated kinase
